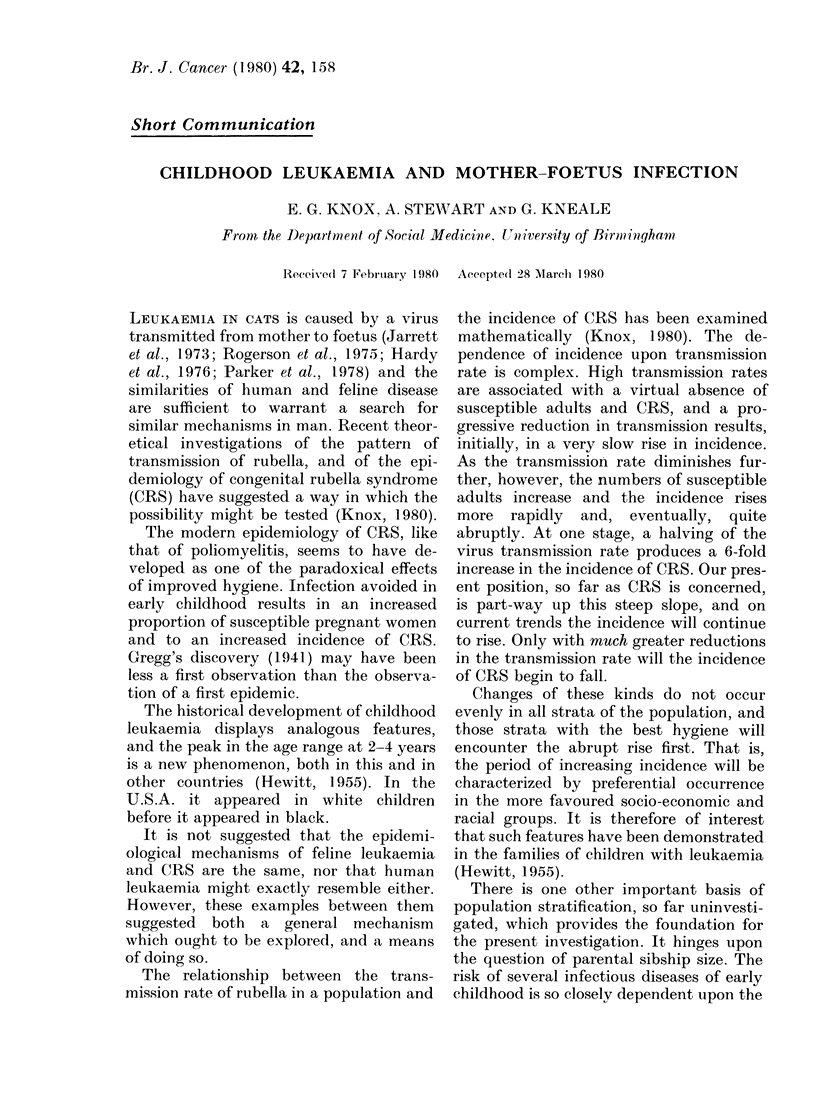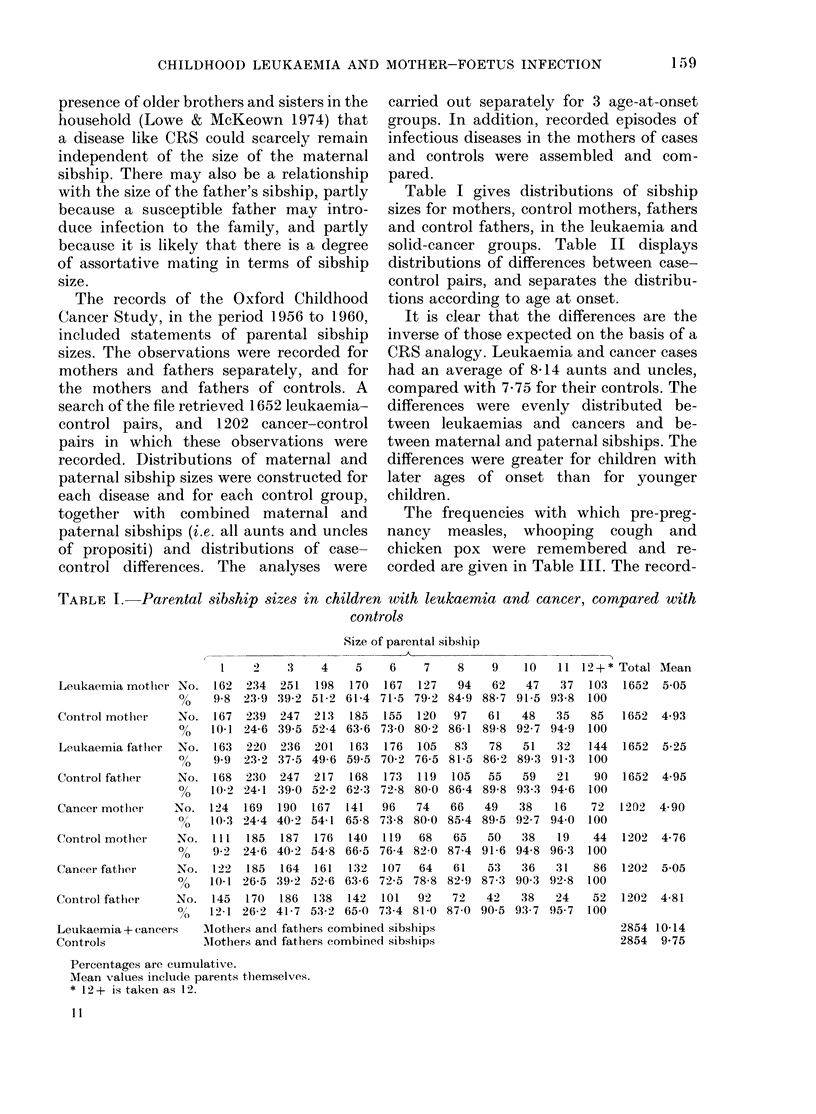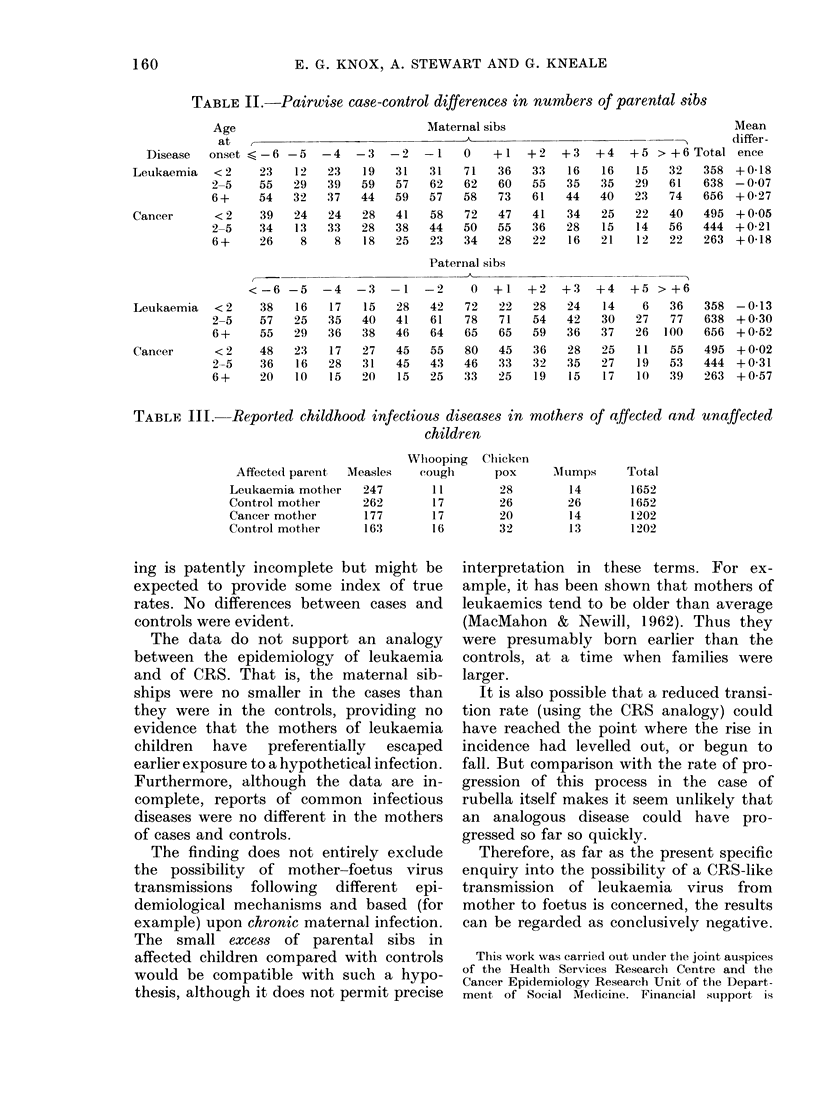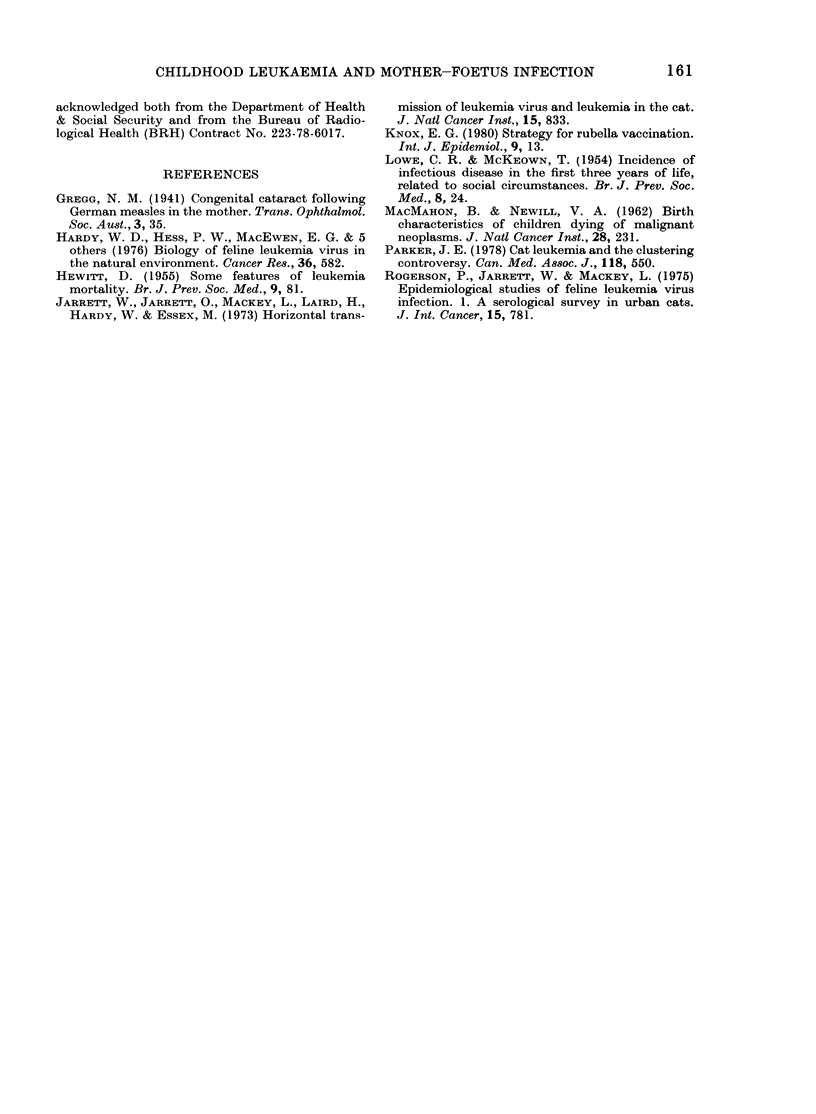# Childhood leukaemia and mother-foetus infection.

**DOI:** 10.1038/bjc.1980.215

**Published:** 1980-07

**Authors:** E. G. Knox, A. Stewart, G. Kneale


*Br. J. Cancer* (1980) **42**, 158

### *Short Communication*

### CHILDHOOD LEUKAEMIA AND MOTHER–FOETUS INFECTION

E. G. KNOX, A. STEWART AND G. KNEALE

*From the Department of Social Medicine, University of Birmingham*

Received 7 February 1980 Accepted 28 March 1980

LEUKAEMIA IN CATS is caused by a virus transmitted from mother to foetus (Jarrett *et al.*, 1973; Rogerson *et al.*, 1975; Hardy *et al.*, 1976; Parker *et al.*, 1978) and the similarities of human and feline disease are sufficient to warrant a search for similar mechanisms in man. Recent theoretical investigations of the pattern of transmission of rubella, and of the epidemiology of congenital rubella syndrome (CRS) have suggested a way in which the possibility might be tested (Knox, 1980).

The modern epidemiology of CRS, like that of poliomyelitis, seems to have developed as one of the paradoxical effects of improved hygiene. Infection avoided in early childhood results in an increased proportion of susceptible pregnant women and to an increased incidence of CRS. Gregg's discovery (1941) may have been less a first observation than the observation of a first epidemic.

The historical development of childhood leukaemia displays analogous features, and the peak in the age range at 2–4 years is a new phenomenon, both in this and in other countries (Hewitt, 1955). In the U.S.A. it appeared in white children before it appeared in black.

It is not suggested that the epidemiological mechanisms of feline leukaemia and CRS are the same, nor that human leukaemia might exactly resemble either. However, these examples between them suggested both a general mechanism which ought to be explored, and a means of doing so.

The relationship between the transmission rate of rubella in a population and the incidence of CRS has been examined mathematically (Knox, 1980). The dependence of incidence upon transmission rate is complex. High transmission rates are associated with a virtual absence of susceptible adults and CRS, and a progressive reduction in transmission results, initially, in a very slow rise in incidence. As the transmission rate diminishes further, however, the numbers of susceptible adults increase and the incidence rises more rapidly and, eventually, quite abruptly. At one stage, a halving of the virus transmission rate produces a 6-fold increase in the incidence of CRS. Our present position, so far as CRS is concerned, is part-way up this steep slope, and on current trends the incidence will continue to rise. Only with *much* greater reductions in the transmission rate will the incidence of CRS begin to fall.

Changes of these kinds do not occur evenly in all strata of the population, and those strata with the best hygiene will encounter the abrupt rise first. That is, the period of increasing incidence will be characterized by preferential occurrence in the more favoured socio-economic and racial groups. It is therefore of interest that such features have been demonstrated in the families of children with leukaemia (Hewitt, 1955).

There is one other important basis of population stratification, so far uninvestigated, which provides the foundation for the present investigation. It hinges upon the question of parental sibship size. The risk of several infectious diseases of early childhood is so closely dependent upon the presence of older brothers and sisters in the household (Lowe & McKeown 1974) that a disease like CRS could scarcely remain independent of the size of the maternal sibship. There may also be a relationship with the size of the father's sibship, partly because a susceptible father may introduce infection to the family, and partly because it is likely that there is a degree of assortative mating in terms of sibship size.

The records of the Oxford Childhood Cancer Study, in the period 1956 to 1960, included statements of parental sibship sizes. The observations were recorded for mothers and fathers separately, and for the mothers and fathers of controls. A search of the file retrieved 1652 leukaemia–control pairs, and 1202 cancer–control pairs in which these observations were recorded. Distributions of maternal and paternal sibship sizes were constructed for each disease and for each control group, together with combined maternal and paternal sibships (*i.e.* all aunts and uncles of propositi) and distributions of case–control differences. The analyses were carried out separately for 3 age-at-onset groups. In addition, recorded episodes of infectious diseases in the mothers of cases and controls were assembled and compared.

Table I gives distributions of sibship sizes for mothers, control mothers, fathers and control fathers, in the leukaemia and solid-cancer groups. Table II displays distributions of differences between case–control pairs, and separates the distributions according to age at onset.

It is clear that the differences are the inverse of those expected on the basis of a CRS analogy. Leukaemia and cancer cases had an average of 8·14 aunts and uncles, compared with 7·75 for their controls. The differences were evenly distributed between leukaemias and cancers and between maternal and paternal sibships. The differences were greater for children with later ages of onset than for younger children.

The frequencies with which pre-pregnancy measles, whooping cough and chicken pox were remembered and recorded are given in Table III. The record-

TABLE I.—*Parental sibship sizes in children with leukaemia and cancer, compared with controls*

| | | Size of parental sibship | | | | | | | | | | | | | |
|---|---|---|---|---|---|---|---|---|---|---|---|---|---|---|---|
| | | 1 | 2 | 3 | 4 | 5 | 6 | 7 | 8 | 9 | 10 | 11 | 12+* | Total | Mean |
| Leukaemia mother | No. | 162 | 234 | 251 | 198 | 170 | 167 | 127 | 94 | 62 | 47 | 37 | 103 | 1652 | 5·05 |
| | % | 9·8 | 23·9 | 39·2 | 51·2 | 61·4 | 71·5 | 79·2 | 84·9 | 88·7 | 91·5 | 93·8 | 100 | | |
| Control mother | No. | 167 | 239 | 247 | 213 | 185 | 155 | 120 | 97 | 61 | 48 | 35 | 85 | 1652 | 4·93 |
| | % | 10·1 | 24·6 | 39·5 | 52·4 | 63·6 | 73·0 | 80·2 | 86·1 | 89·8 | 92·7 | 94·9 | 100 | | |
| Leukaemia father | No. | 163 | 220 | 236 | 201 | 163 | 176 | 105 | 83 | 78 | 51 | 32 | 144 | 1652 | 5·25 |
| | % | 9·9 | 23·2 | 37·5 | 49·6 | 59·5 | 70·2 | 76·5 | 81·5 | 86·2 | 89·3 | 91·3 | 100 | | |
| Control father | No. | 168 | 230 | 247 | 217 | 168 | 173 | 119 | 105 | 55 | 59 | 21 | 90 | 1652 | 4·95 |
| | % | 10·2 | 24·1 | 39·0 | 52·2 | 62·3 | 72·8 | 80·0 | 86·4 | 89·8 | 93·3 | 94·6 | 100 | | |
| Cancer mother | No. | 124 | 169 | 190 | 167 | 141 | 96 | 74 | 66 | 49 | 38 | 16 | 72 | 1202 | 4·90 |
| | % | 10·3 | 24·4 | 40·2 | 54·1 | 65·8 | 73·8 | 80·0 | 85·4 | 89·5 | 92·7 | 94·0 | 100 | | |
| Control mother | No. | 111 | 185 | 187 | 176 | 140 | 119 | 68 | 65 | 50 | 38 | 19 | 44 | 1202 | 4·76 |
| | % | 9·2 | 24·6 | 40·2 | 54·8 | 66·5 | 76·4 | 82·0 | 87·4 | 91·6 | 94·8 | 96·3 | 100 | | |
| Cancer father | No. | 122 | 185 | 164 | 161 | 132 | 107 | 64 | 61 | 53 | 36 | 31 | 86 | 1202 | 5·05 |
| | % | 10·1 | 26·5 | 39·2 | 52·6 | 63·6 | 72·5 | 78·8 | 82·9 | 87·3 | 90·3 | 92·8 | 100 | | |
| Control father | No. | 145 | 170 | 186 | 138 | 142 | 101 | 92 | 72 | 42 | 38 | 24 | 52 | 1202 | 4·81 |
| | % | 12·1 | 26·2 | 41·7 | 53·2 | 65·0 | 73·4 | 81·0 | 87·0 | 90·5 | 93·7 | 95·7 | 100 | | |
| Leukaemia + cancers | Mothers and fathers combined sibships | | | | | | | | | | | | | 2854 | 10·14 |
| Controls | Mothers and fathers combined sibships | | | | | | | | | | | | | 2854 | 9·75 |

Percentages are cumulative.
Mean values include parents themselves.
* 12+ is taken as 12.

TABLE II.—*Pairwise case-control differences in numbers of parental sibs*

| Disease | Age at onset | Maternal sibs ⩽ −6 | −5 | −4 | −3 | −2 | −1 | 0 | +1 | +2 | +3 | +4 | +5 | > +6 | Total | Mean difference |
|---|---|---|---|---|---|---|---|---|---|---|---|---|---|---|---|---|
| Leukaemia | < 2 | 23 | 12 | 23 | 19 | 31 | 31 | 71 | 36 | 33 | 16 | 16 | 15 | 32 | 358 | +0·18 |
| | 2–5 | 55 | 29 | 39 | 59 | 57 | 62 | 62 | 60 | 55 | 35 | 35 | 29 | 61 | 638 | −0·07 |
| | 6+ | 54 | 32 | 37 | 44 | 59 | 57 | 58 | 73 | 61 | 44 | 40 | 23 | 74 | 656 | +0·27 |
| Cancer | < 2 | 39 | 24 | 24 | 28 | 41 | 58 | 72 | 47 | 41 | 34 | 25 | 22 | 40 | 495 | +0·05 |
| | 2–5 | 34 | 13 | 33 | 28 | 38 | 44 | 50 | 55 | 36 | 28 | 15 | 14 | 56 | 444 | +0·21 |
| | 6+ | 26 | 8 | 8 | 18 | 25 | 23 | 34 | 28 | 22 | 16 | 21 | 12 | 22 | 263 | +0·18 |
| | | Paternal sibs < −6 | −5 | −4 | −3 | −1 | −2 | 0 | +1 | +2 | +3 | +4 | +5 | > +6 | | |
| Leukaemia | < 2 | 38 | 16 | 17 | 15 | 28 | 42 | 72 | 22 | 28 | 24 | 14 | 6 | 36 | 358 | −0·13 |
| | 2–5 | 57 | 25 | 35 | 40 | 41 | 61 | 78 | 71 | 54 | 42 | 30 | 27 | 77 | 638 | +0·30 |
| | 6+ | 55 | 29 | 36 | 38 | 46 | 64 | 65 | 65 | 59 | 36 | 37 | 26 | 100 | 656 | +0·52 |
| Cancer | < 2 | 48 | 23 | 17 | 27 | 45 | 55 | 80 | 45 | 36 | 28 | 25 | 11 | 55 | 495 | +0·02 |
| | 2–5 | 36 | 16 | 28 | 31 | 45 | 43 | 46 | 33 | 32 | 35 | 27 | 19 | 53 | 444 | +0·31 |
| | 6+ | 20 | 10 | 15 | 20 | 15 | 25 | 33 | 25 | 19 | 15 | 17 | 10 | 39 | 263 | +0·57 |

TABLE III.—*Reported childhood infectious diseases in mothers of affected and unaffected children*

| Affected parent | Measles | Whooping cough | Chicken pox | Mumps | Total |
|---|---|---|---|---|---|
| Leukaemia mother | 247 | 11 | 28 | 14 | 1652 |
| Control mother | 262 | 17 | 26 | 26 | 1652 |
| Cancer mother | 177 | 17 | 20 | 14 | 1202 |
| Control mother | 163 | 16 | 32 | 13 | 1202 |

ing is patently incomplete but might be expected to provide some index of true rates. No differences between cases and controls were evident.

The data do not support an analogy between the epidemiology of leukaemia and of CRS. That is, the maternal sibships were no smaller in the cases than they were in the controls, providing no evidence that the mothers of leukaemia children have preferentially escaped earlier exposure to a hypothetical infection. Furthermore, although the data are incomplete, reports of common infectious diseases were no different in the mothers of cases and controls.

The finding does not entirely exclude the possibility of mother–foetus virus transmissions following different epidemiological mechanisms and based (for example) upon *chronic* maternal infection. The small *excess* of parental sibs in affected children compared with controls would be compatible with such a hypothesis, although it does not permit precise interpretation in these terms. For example, it has been shown that mothers of leukaemics tend to be older than average (MacMahon & Newill, 1962). Thus they were presumably born earlier than the controls, at a time when families were larger.

It is also possible that a reduced transition rate (using the CRS analogy) could have reached the point where the rise in incidence had levelled out, or begun to fall. But comparison with the rate of progression of this process in the case of rubella itself makes it seem unlikely that an analogous disease could have progressed so far so quickly.

Therefore, as far as the present specific enquiry into the possibility of a CRS-like transmission of leukaemia virus from mother to foetus is concerned, the results can be regarded as conclusively negative.

This work was carried out under the joint auspices of the Health Services Research Centre and the Cancer Epidemiology Research Unit of the Department of Social Medicine. Financial support is acknowledged both from the Department of Health & Social Security and from the Bureau of Radiological Health (BRH) Contract No. 223-78-6017.

### REFERENCES

GREGG, N. M. (1941) Congenital cataract following German measles in the mother. *Trans. Ophthalmol. Soc. Aust.*, **3**, 35.

HARDY, W. D., HESS, P. W., MACEWEN, E. G. & 5 others (1976) Biology of feline leukemia virus in the natural environment. *Cancer Res.*, **36**, 582.

HEWITT, D. (1955) Some features of leukemia mortality. *Br. J. Prev. Soc. Med.*, **9**, 81.

JARRETT, W., JARRETT, O., MACKEY, L., LAIRD, H., HARDY, W. & ESSEX, M. (1973) Horizontal transmission of leukemia virus and leukemia in the cat. *J. Natl Cancer Inst.*, **15**, 833.

KNOX, E. G. (1980) Strategy for rubella vaccination. *Int. J. Epidemiol.*, **9**, 13.

LOWE, C. R. & MCKEOWN, T. (1954) Incidence of infectious disease in the first three years of life, related to social circumstances. *Br. J. Prev. Soc. Med.*, **8**, 24.

MACMAHON, B. & NEWILL, V. A. (1962) Birth characteristics of children dying of malignant neoplasms. *J. Natl Cancer Inst.*, **28**, 231.

PARKER, J. E. (1978) Cat leukemia and the clustering controversy. *Can. Med. Assoc. J.*, **118**, 550.

ROGERSON, P., JARRETT, W. & MACKEY, L. (1975) Epidemiological studies of feline leukemia virus infection. 1. A serological survey in urban cats. *J. Int. Cancer*, **15**, 781.